# Retraction Note: LINC01089 functions as a ceRNA for miR-152-3p to inhibit non‐small lung cancer progression through regulating PTEN

**DOI:** 10.1186/s12935-022-02830-5

**Published:** 2022-12-15

**Authors:** Huixian Zhang, Hao Zhang, Xingya Li, Siyuan Huang, Qianqian Guo, Di Geng

**Affiliations:** 1 Department of Oncology, The First Affiliated Hospital of Zhengzhou University, Νo.1 Jianshe East Road, Zhengzhou, 450052 Henan China; 2 Department of Radiology, Changhai Hospital, The Second Military Medical University, No.168 Changhai Road, Shanghai, 200433 China

**Retraction: Cancer Cell International (2021) 21:143** **https://doi.org/10.1186/s12935-021-01846-7**

The Editors in Chief have retracted this article. After publication concerns were raised regarding the expression ranges of LINC01089 in the tumour group in Fig. 1C and in Fig. 3B, an evaluation of the raw data found inconsistencies between the provided data and the data in the graphs. Concerns were also raised regarding irregularities within Fig. 5E, however the authors were unable to provide the full original raw western blot data. The Editors in Chief, therefore, no longer have confidence in the reliability of the results presented in this article. None of the authors have responded to any correspondence from the editor/publisher about this retraction.

